# Genome-wide characterization and comparative analysis of R2R3-MYB transcription factors shows the complexity of MYB-associated regulatory networks in *Salvia miltiorrhiza*

**DOI:** 10.1186/1471-2164-15-277

**Published:** 2014-04-11

**Authors:** Caili Li, Shanfa Lu

**Affiliations:** 1 Institute of Medicinal Plant Development, Chinese Academy of Medical Sciences & Peking Union Medical College, No.151, Malianwa North Road, Haidian District, Beijing 100193, China

## Abstract

**Background:**

*MYB* is the largest plant transcription factor gene family playing vital roles in plant growth and development. However, it has not been systematically studied in *Salvia miltiorrhiza*, an economically important medicinal plant.

**Results:**

Here we report the genome-wide identification and characterization of 110 *R2R3*-*MYBs*, the largest subfamily of *MYBs* in *S. miltiorrhiza*. The MYB domain and other motifs of SmMYBs are largely conserved with *Arabidopsis* AtMYBs, whereas the divergence of SmMYBs and AtMYBs also exists, suggesting the conservation and diversity of plant *MYBs*. SmMYBs and AtMYBs may be classified into 37 subgroups, of which 31 include proteins from *S. miltiorrhiza* and *Arabidopsis*, whereas 6 are specific to a species, indicating that the majority of MYBs play conserved roles, while others may exhibit species-specialized functions. *SmMYBs* are differentially expressed in various tissues of *S. miltiorrhiza*. The expression profiles are largely consistent with known functions of their *Arabidopsis* counterparts. The expression of a subset of *SmMYBs* is regulated by microRNAs, such as miR159, miR319, miR828 and miR858. Based on functional conservation of MYBs in a subgroup, SmMYBs potentially involved in the biosynthesis of bioactive compounds were identified.

**Conclusions:**

A total of 110 *R2R3*-*MYBs* were identified and analyzed. The results suggest the complexity of *MYB*-mediated regulatory networks in *S. miltiorrhiza* and provide a foundation for understanding the regulatory mechanism of *SmMYBs*.

## Background

*Salvia* is characterized with only two stamens connected to form a lever. It includes about 900 species and is the largest genus of the Labiatae family. *Salvia*, together with the genera *Lepechinia*, *Melissa*, *Dorystaechas*, *Meriandra*, *Zhumeria*, *Perovskia* and *Rosmarinus*, forms a monophylectic lineage within the Labiatae [1,2]. *S. miltiorrhiza* Bunge, known as Danshen in Chinese, is an economically important medicinal plant species of the *Salvia* genus. It shows close phylogenetic relationships with other Asian and Mediterranean species in the *Salvia* genus, such as *S. roborowskii* and *S. glutinosa*[2]. *S. miltiorrhiza* has been widely and successfully used in traditional Chinese medicine (TCM) for hundred of years to treat numerous diseases, such as coronary heart diseases, dysmenorrheal, amenorrhoea, and inflammatory diseases [3,4]. The main bioactive components of *S. miltiorrhiza* may be divided into two groups. The first group is abietane type-diterpene quinine pigments, known as tanshinones, which are lipophilic and consist of more than thirty compounds [5]. The second group is hydrophilic phenolic acids, including rosmarinic acid, salvianolic acid A, salvianolic acid B, lithospermic acid, and many other chemicals [6]. Biosynthesis of main bioactive components in *S. miltiorrhiza* requires the coordination of a series of key enzymes [7]. The expression of genes encoding these key enzymes is regulated by various transcription factors, of which MYBs appear to play significant roles [8,9]. Identification and characterization of *MYB* genes in *S. miltiorrhiza* is very important in understanding the regulatory mechanism of bioactive component biosynthesis. Since MYBs are also vital regulators in plant development and plant responses to various biotic and abiotic stresses, elucidation of MYB-associated regulatory networks may greatly help in improving the growth and defense abilities of *S. miltiorrhiza* through genetic engineering approaches.

MYB proteins, characterized by the MYB domain, have been widely found in many organisms, including animals, fungi and plants. The MYB domain of MYB proteins is deeply conserved and contains up to four imperfect repeats (R) of about 52 amino acids [10]. Many vertebrates contain three *MYB* genes, known as *c*-*MYB*, *A*-*MYB* and *B*-*MYB*[11]. The repeats in c-MYB proteins are referred to as R1, R2 and R3. Repeats from other proteins are named based on their similarity to R1, R2 or R3 of c-MYB proteins [10,12]. In plants, MYB is the largest transcription factor family and may be classified into four subfamilies based on the number of adjacent imperfect repeats [10,13]. It includes R1R2R3R1/2-MYBs (4R-MYBs), R1R2R3-MYBs (3R-MYBs), R2R3-MYBs and MYBs with a single or partial MYB repeat (1R-MYB or MYB-related), of which R2R3-MYB is the largest subfamily of plant MYBs [10]. The number of *R2R3*-*MYB* genes in *Arabidopsis thaliana*, *Populus trichocarpa* and *Oryza sativa* is 137, 198, and 95, respectively [13].

R2R3-MYB proteins contain two repeats similar to R2 and R3 of c-MYB proteins and have been proposed to be evolved from a 3R-MYB gene ancestor by the loss of R1 [14]. In addition to the MYB domain, other less conserved motifs were also found in R2R3-MYB proteins. Based on the similarity of these motifs or phylogenetic relationships of MYB amino acids, R2R3-MYBs may be divided into subgroups. For instance, the large *Arabidopsis* R2R3-MYB subfamily has been divided into 25 subgroups according to the motifs [10,12]. However, based on phylogenetic relationships, the number of R2R3-MYB subgroups in *Arabidopsis*, *P. trichocarpa*, soybean and maize is 40, 42, 48, and 18, respectively [15-17]. It suggests that the resulting number of R2R3-MYB subgroups in *Arabidopsis* significantly varies between two approaches used and shows that some subgroups are not deeply conserved among different plant species. Proteins in non-conserved subgroups might have specialized roles.

The functions of numerous R2R3-MYBs have been characterized in *Arabidopsis*. The known functions were well-summarized into four plant-specific processes, including primary and secondary metabolism, cell fate and identity, developmental processes and responses to biotic and abiotic stresses [10]. It clearly demonstrates functional divergence and significance of *Arabidopsis* R2R3-MYBs. Extensive studies on the *MYB* gene family have been conducted in various plant species, such as rice, maize, wheat, poplar, eucalyptus and gentian [18-23]. The results showed that the functions of R2R3-MYBs belonging to the same subgroup were usually conserved among different plant species. For instance, subgroup 7 MYBs, including *Arabidopsis* AtMYB11, AtMYB12 and AtMYB111 [24], grape VvMYBF1 [25], tomato SlMYB12 [26], gentian GtMYBP3 and GtMYBP4 [27], and many others, are involved in the control of flavonol biosynthesis. Subgroup 6 MYBs, such as *Arabidopsis* AtMYB75/PAP1, AtMYB90/PAP2, AtMYB113 and AtMYB114 [28], cauliflower BoMYB2 [29], apple MYB110a [30], and pear PyMYB10 [31], regulate anthocyanin biosynthesis. It suggests that the functions of MYBs may be predicted by phylogenetic analysis. So far, little is known about MYBs in *S. miltiorrhiza*.

*S. miltiorrhiza* is emerging as a model plant for TCM studies, since its relatively small genome size (~600 Mb), short life cycle, undemanding growth requirements, and significant medicinal value [7]. Recently, the genome of *S. miltiorrhiza* has been sequenced and a working draft of the genome has been assembled (Chen et al., unpublished). In order to elucidate the role of *MYB* genes in *S. miltiorrhiza* development, defense and bioactive component biosynthesis, we performed a genome-wide analysis of the *R2R3*-*MYB* gene subfamily in *S. miltiorrhiza*.

## Results and discussion

### Identification of 110 *S. miltiorrhiza* R2R3-MYB genes

Through BLAST analysis of 125 *A. thaliana* R2R3-MYBs [12] against the current assembly of the *S. miltiorrhiza* genome (Chen et al., unpublished) and subsequent gene prediction of the retrieved genomic DNA sequences, a total of 110 full-length or near full-length members of the *S. miltiorrhiza R2R3*-*MYB* gene subfamily were predicted. Notably, it may not be a complete set of *R2R3*-*MYB* genes in *S. miltiorrhiza*, since the current assembly of the *S. miltiorrhiza* genome is just a working draft and we do identified from the current assembly some partial sequences with high homology to known *R2R3*-*MYBs* in other plant species (data not shown). To verify the results from computational prediction,primers were designed for PCR-amplification of full-length coding sequences (CDSs) of 110 *S. miltiorrhiza R2R3*-*MYBs*. A total of 109 CDSs were obtained. Sequence comparison showed that the cloned CDSs of 102 *R2R3*-*MYBs* were identical to the predicted sequences. The other seven cloned CDSs, including those of *SmMYB1*, *SmMYB13*, *SmMYB16*, *SmMYB35*, *SmMYB62*, *SmMYB63* and *SmMYB79*, differed from the predicted ones by one or a few bases only; However, the deduced amino acids were identical. The results confirm the correctness of *MYB* gene annotation and suggest the reliability of DNA sequence data. We were not able to clone the CDS of *SmMYB108*. It could be due to low expression level in the tissues analyzed. All 109 cloned CDSs and the predicted *SmMYB108* have been submitted to GenBank. The accession numbers in GenBank are shown in Additional file 1: Table S1.

### Phylogenetic analysis of R2R3-MYB proteins from *S. miltiorrhiza* and *Arabidopsis*

**Figure 1:**
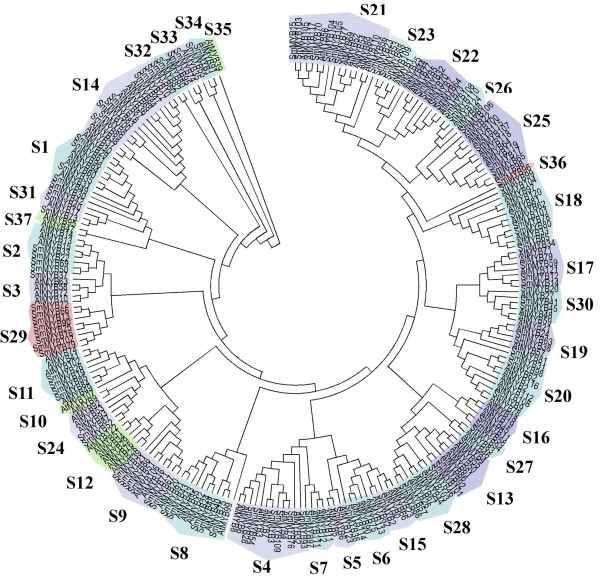
**Phylogenetic relationships of MYB proteins from *S. miltiorrhiza *and *Arabidopsis.*** Subgroups are highlighted.

In order to know the relationship of R2R3-MYBs in *S. miltiorrhiza* and *Arabidopsis*, a neighbor-joining (NJ) phylogenetic tree was constructed using MEGA4.0 (Figure 1). The results showed that many *S. miltiorrhiza MYBs* were highly similar to their counterparts in *Arabidopsis*. Based on the phylogenetic tree and previous results from *Arabidopsis*[10], R2R3-MYBs in *S. miltiorrhiza* and *Arabidopsis* might be classified into 37 subgroups (named S1–S37), of which S1–S25 were named as previously described [10]; while the others were novel. Thirty one of 34 subgroups included proteins from *S. miltiorrhiza* and *Arabidopsis*, whereas the other six were specific to *S. miltiorrhiza* (S29 and S36) or *Arabidopsis* (S10, S12, S35 and S37). Species-specific subgroups of R2R3-MYBs have also been found in other plant species, such as rice [16], maize [17] and wheat [32]. Since MYBs in a subgroup usually play similar roles or function in a metabolic pathway [10], our results indicate that some MYBs play deeply conserved roles in *S. miltiorrhiza* and *Arabidopsis*, while the others may exhibit species-specialized functions.

### Conservation and divergence of MYB domain

**Figure 2:**
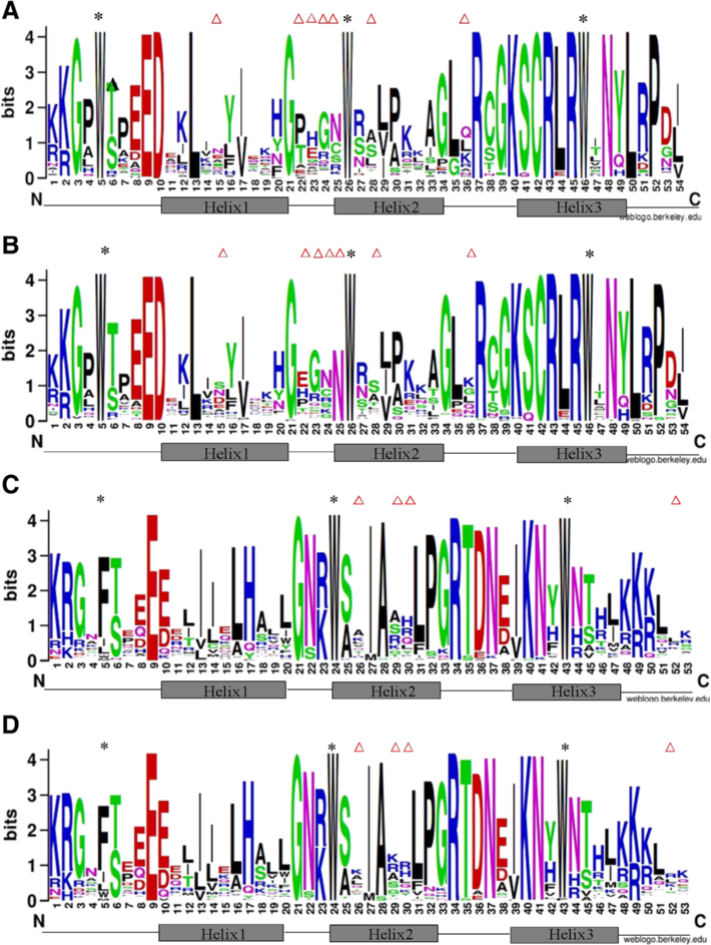
**Comparison of R2 and R3 sequences in R2R3-MYBs from *S. miltiorrhiza *and *Arabidopsis. *(A)** and **(B)** Sequence logos of R2 in MYBs from *S. miltiorrhiza***(A)** and *Arabidopsis***(B)**. **(C)** and **(D)** Sequence logos of R3 in MYBs from *S. miltiorrhiza***(C)** and *Arabidopsis***(D)**. Bits represent the conservation of sequence at a position. The positions with different patterns between *S. miltiorrhiza* and *Arabidopsis* are indicated by triangles. Highly conserved tryptophan (W) and phenylalanine (F) residues are indicated by asterisks.

The MYB domain of MYB proteins is highly conserved in plants. It contains up to four imperfect repeats (R) of about 52 amino acids [10]. R2R3-MYBs are characterized with two repeats, known as R2 and R3. Consistently, the MYB domain of *S. miltiorrhiza* and *Arabidopsis* R2R3-MYBs contain 107 residues, of which 54 form R2, while the other 53 constitute R3 (Figure 2). In order to elucidate sequence features of MYB domain and the degree of conservation of each residue, multiple sequence alignment was performed and sequence logos were created for R2 and R3 of R2R3-MYBs from *S. miltiorrhiza* and *Arabidopsis* (Figure 2). The results showed that the distribution of residues in R2 and R3 of *S. miltiorrhiza* R2R3-MYBs was quite similar to *Arabidopsis* (Figure 2B and Figure 2D). R2 of both *S. miltiorrhiza* and *Arabidopsis* R2R3-MYBs contains three highly conserved tryptophan residues (W) at positions 5, 26 and 46 (Figure 2A and Figure 2B), which may form a tryptophan cluster in the 3-dimensional HTH structure and play significant roles in MYB-DNA interaction [15,17]. Similarly, three regularly spaced and highly conserved residues, including a phenylalanine (F) and two tryptophan residues, exist at positions 5, 24 and 43 of R3 (Figure 2C and Figure 2D). The highly conserved tryptophan residues were also found in R2R3-MYBs from other plant species, such as *Populus trichocarpa*[15] and soybean [16]. Although the phenylalanine at position 5 of R3 is conserved in R2R3-MYBs from *S. miltiorrhiza*, *Arabidopsis*, and other plant species, such as *P. trichocarpa*[15] and soybean [16], the degree of conservation is apparently less compared with the tryptophan residues at positions 24 and 43 (Figure 2C and Figure 2D). The functional significance of phenylalanine at position 5 of R3 remains to be elucidated. The other conserved residues in R2 and R3 were mainly distributed between the second and the third residues of three conserved residues described above (Figure 2). The residues between the first and the second conserved tryptophan in R2 and those between the conserved phenylalanine and the first conserved tryptophan in R3, particularly the residues forming the first helix in each repeat, are apparently less conserved (Figure 2). The 3’ region of R2 in both *S. miltiorrhiza* and *Arabidopsis* R2R3-MYBs contains a highly conserved LRPD motif (LRPD), which was also observed in *P. trichocarpa*[15], soybean [16] and maize [17]. The results suggest the conservation of amino acid distribution in the MYB domain of plant R2R3-MYBs. On the other hand, the patterns at positions 15, 22–25, 28 and 36 of R2 and 26, 29, 30 and 52 of R3 are different between *S. miltiorrhiza* and *Arabidopsis* (Figure 2), showing divergence of the MYB domain.

### Analysis of conserved motifs other than the MYB domain

**Figure 3:**
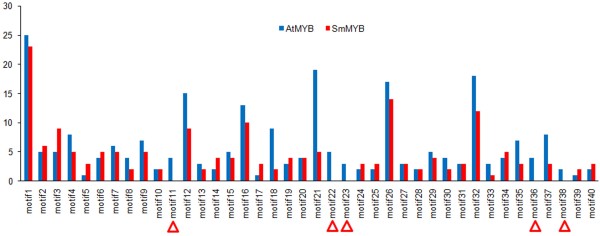
**The number of *S. miltiorrhiza *and *Arabidopsis *R2R3-MYB proteins containing conserved motifs outside the MYB domain.** Five AtMYB-specific motifs are indicated by triangles.

**Figure 4:**
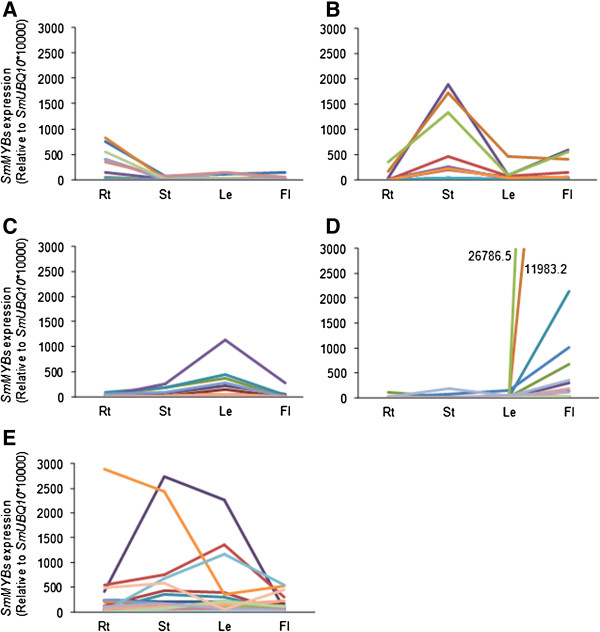
**Clustering of differentially expressed *MYB *genes in roots (Rt), stems (St), leaves (Le) and flowers (Fl) of *S. miltiorrhiza. *(A)** Ten *SmMYBs* predominantly expressed in roots. **(B)** Twelve *SmMYBs* predominantly expressed in stems. **(C)** Thirty one *SmMYBs* predominantly expressed in leaves. **(D)** Seventeen *SmMYBs* predominantly expressed in flowers. **(E)** Thirty nine *SmMYBs* highly expressed in at least two tissues analyzed. The expression level of each gene is relative to SmUBQ10*10000.

It has been shown that the C-terminal region next to the MYB domain of R2R3-MYBs usually contains functionally important motifs, although these motifs are less conserved compared with the MYB domain [10,12]. Using the MEME suite, a total of 40 motifs were identified in the downstream of MYB domain of *S. miltiorrhiza* and *Arabidopsis* R2R3-MYBs (Figure 3 and Additional file 2: Figure S1). The length of motifs varies from 8 to 107 amino acids and the number of motifs in each MYB varies between 0 and 6. No motifs were predicted for 14 SmMYBs and 10 AtMYBs. Although the majority of 40 motifs exist in both *S. miltiorrhiza* and *Arabidopsis* R2R3-MYBs, five (motifs 11, 22, 23, 36 and 38) are AtMYB-specific (Figure 4). No SmMYB-specific motifs were identified. Among 40 motifs, motif 1 is the most common motif, which was found in 25 AtMYBs and 23 SmMYBs. The next common motifs are motifs 26 and 32 present in 17 AtMYBs/14 SmMYBs and 18 AtMYBs/12 SmMYBs, respectively. Many *S. miltiorrhiza* R2R3-MYBs in a subgroup share at least a motif. Consistently, many *Arabidoopsis* R2R3-MYBs in a subgroup contain same motif(s) as their *S. miltiorrhiza* orthologues in the subgroup (Additional file 2: Figure S1) [10,12]. It suggests the conservation of motifs in *S. miltiorrhiza* and *Arabidopsis* R2R3-MYBs belonging to a subgroup. The majority of motifs were found in more than one subgroup of R2R3-MYBs, except motifs 8 and 29 existing in S1 and motif 18 in S18. For instance, motifs 21 and 26 widely exist in R2R3-MYBs belonging to 16 and 15 subgroups, respectively (Additional file 2: Figure S1). Taken together, the results suggest that these motifs are evolutionarily conserved and functionally important; however, it is currently unknown for the underlying mechanism of motifs to be under selection and conserved among divergent species.

### Expression profiling of *S. miltiorrhiza R2R3*-*MYB* genes

**Figure 5:**
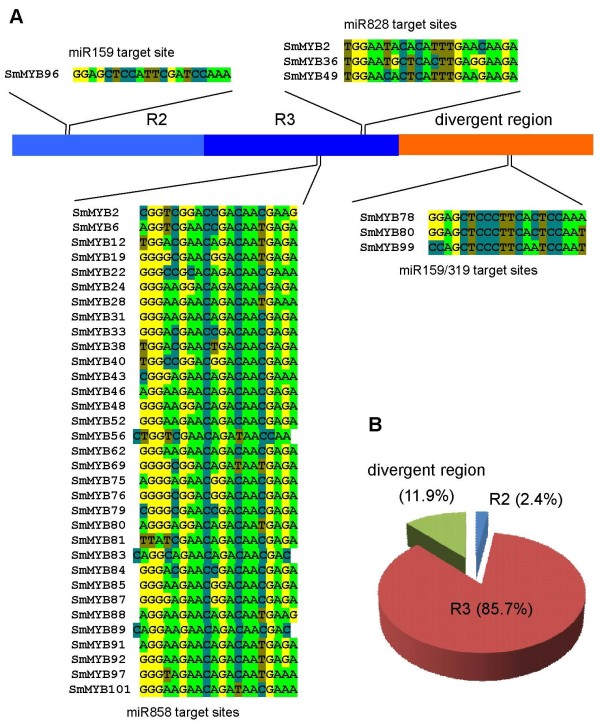
***SmMYBs *targeted by miR858, miR828 and miR159/319. (A)** miRNA-targeted *SmMYB* sequences and their locations. **(B)** Proportion of target sites located in R2, R3 and the divergent region.

In order to elucidate possible roles of *R2R3*-*MYBs* in the growth and development of *S. miltiorrhiza*, we investigated the relative expression level of 110 *SmMYBs* in roots, stems, leaves and flowers of two-year-old, field nursery-grown *S. miltiorrhiza* plants using the quantitative real-time RT-PCR method. Transcripts were detected for 109 of 110 *SmMYBs* (Figure 4 and Additional file 3: Figure S2). The expression of *SmMYB108* was undetected, which is consistent with the result from CDS cloning. It indicates that *SmMYB108* may be pseudogenes or expressed at specific developmental stages or under special conditions. Of the 109 detectable *SmMYBs*, 31 (28%) showed predominant expression in leaves, 17 (15.5%) in flowers, 12 (11%) in stems, and 10 (9%) in roots. The other 39 (35.5%) were highly expressed in at least two tissues analyzed (Figure 5). Differential expression of *SmMYBs* is consistent with the fact that each *MYB* is usually involved in a limited number of cellular processes.

Further examination of the expression of *SmMYBs* in each subgroup showed that some *MYB* genes in a subgroup shared similar expression profiles and the profiles were consistent with known functions of their *Arabidopsis* counterparts. For instance, S18 contains 4 SmMYBs and 7 AtMYBs (Figure 2), of which AtMYB33 and AtMYB65 are gibberellin-regulated MYBs (GAMYBs) involved in gibberellin signaling [33-41]. It has been shown that *AtMYB33* and *AtMYB65* redundantly facilitate anther development. In the anther of a *myb33 myb65* double mutant, the tapetum underwent hypertrophy at the pollen mother cell stage [41]. Analyzing the expression of *AtMYB33* and *AtMYB65* showed complete repression in vegetative tissues through a miR159-associated regulatory mechanism [42]. Consistently, *SmMYB62*, *SmMYB78*, *SmMYB80* and *SmMYB99*, four *S. miltiorrhiza MYBs* include in S18, were predominantly expressed in flowers (Additional file 3: Figure S2), suggesting their significant roles in flower development. In addition, SmMYB37 belonging to S3 was predominately expressed in *S. miltiorrhiza* stems with plenty of lignin (Additional file 3: Figure S2). It is consistent with the function of AtMYB58 and AtMYB63 belonging to the same subgroup of SmMYB37 in activation of *PAL*, *C4H* and *4CL* genes involved in the phenylpropanoid pathway and various other genes related to lignin biosynthesis [43].

On the other hand, many *SmMYB* genes in a subgroup showed differential expression in *S. miltiorrhiza*. Even so, the expression profiles were still consistent with known functions of their *Arabidopsis* counterparts. S14 includes 9 SmMYBs and 6 AtMYBs, of which SmMYB47 and AtMYB36 were root-specific. SmMYB100 and AtMYB68 was exclusively found in roots and exhibited low level in flowers. AtMYB37 was predominantly expressed in roots and was also found in flowers and shoots. SmMYB18 was highly expressed in stems and less in leaves. SmMYB60 was highly expressed in leaves and less in stems. SmMYB27 was abundant in roots and less in stems and leaves, while SmMYB8, SmMYB10, SmMYB83, SmMYB89, AtMYB38, AtMYB84 and AtMYB87 were expressed in all tissues analyzed (Additional file 3: Figure S2) [44]. Consistently, AtMYB37, AtMYB38 and AtMYB84 redundantly regulate axillary meristem formation at different stages of vegetative development [45,46], whereas AtMYB68 is a root growth-specific regulator [46]. AtMYB16 and AtMYB106 belonging to S9 are MIXTA-like transcription factors involved in trichome differentiation, petal cell development and petal morphogenesis [47-52]. Consistently, all S9 SmMYBs, including SmMYB21, SmMYB39, SmMYB51, SmMYB67, SmMYB71 and SmMYB86, were highly expressed in leaves with abundant of trichomes (Additional file 3: Figure S2). In addition, SmMYB39, SmMYB67 and SmMYB71 were also highly expressed in flowers (Additional file 3: Figure S2). Taken together, the results strongly suggest that many SmMYBs play similar roles as AtMYBs in the same subgroup.

### miRNA-mediated post-transcriptional regulation of *SmMYBs*

Plant microRNAs (miRNAs) are a class of endogenous noncoding RNAs with size about 21 nucleotides. They play vital roles in plant development and stress responses mainly through direct cleavage of target mRNAs with perfect or near-perfect complementarities [53-58]. It allows an effective prediction of target sequence through computational approaches [59]. So far, a total of 190 *Arabidopsis* miRNA gene families, representing 338 mature miRNAs, have been identified [60]. Among them, eight (miR159a, miR159b, miR159c, miR319a, miR319b, miR319c, miR828 and miR858) produced from three gene families, including *MIR159*/*319*, *MIR828* and *MIR858*, were found to regulate the expression of various *AtMYB* genes [10,13,61,62]. The regulatory role of miRNAs in *MYBs* also exists in apple [63]. In order to elucidate miRNA-mediated posttranscriptional regulation of *S. miltiorrhiza MYBs*, we searched all *SmMYBs* for potential targets of deeply conserved miR159, miR319, miR828 and miR858 [64]. A total of 38 *SmMYBs* with complementary sequence to the member of *MIR159*/*319*, *MIR828* and *MIR858* families were identified (Figure 5). Of 38 *SmMYBs*, 31 were predicted targets of miR858, 2 were of miR828, 2 were of miR159, while the other 3 were common targets of miR828 and miR858, miR159 and miR319, or miR159, miR319 and miR858. It suggests the identification of total 42 miRNA target sites, of which 36 (85.7%) locate in R3, 1 in R2, and 5 (11.9%) in other regions (Figure 5). The large proportion of putative miRNA target sites in R3 could be due to the deep conservation of sequence in this region. Although all target sites of miR828 and miR858 locate in R3, miR858 target to more conserved 5’ end, while miR828 targets to less conserved 3’ end. It results in that the number of miR858 targets, 33, is much greater than that of miR828, 3. One of four target sites of miR159 locates in R2. The other three target sites of miR159 and two target sites of miR319, including the overlapping target sites of miR159 and miR319 in *SmMYB78* and *SmMYB80*, locate in less conserved regions other than R2 and R3 (Figure 5). It suggests the conservation and divergence of miRNA target sites and indicates that the number of miRNA targets in a gene family is associated with the location of target sites.

The majority of 38 *SmMYBs* are regulated by a miRNA family, whereas the other two, including *SmMYB2* and *SmMYB80*, are targeted by miRNAs from two families (Figure 1). *SmMYB2*, which is included in S15 and highly expressed in leaves, is a target of the *MIR828* and *MIR858* family members. In S15, there are 4 *Arabidopsis* MYBs, of which AtMYB0, AtMYB23 and AtMYB66 are involved in trichome development [41,65]; however they are not targets of miR828 and miR858. The other S15 *Arabidopsis MYB*, known as AtMYB82, is a potential target of miR828 and miR858. Since functions of AtMYB82 are currently unknown, the significance of miR828- and miR858-involved co-regulation remains to be elucidated. Similarly, *SmMYB80* belonging to S18 and predominantly expressed in flowers is co-regulated by members of *MIR159*/*319* and *MIR858*. Further analyzing the regulation role of miRNAs in 4 SmMYBs and 7 AtMYBs included in S18 showed that all of them could be targets of miRNAs. Of 11 MYBs, SmMYB99 and AtMYB101 are regulated by miR159. SmMYB62 and AtMYB81 are targets of miR858. SmMYB78 and AtMYB33 are targets of miR159 and miR319. AtMYB97 and AtMYB120 are targets of miR159 and miR858. SmMYB80 and AtMYB65 and AtMYB104 are regulated by miR159 and miR319 and miR858. All SmMYBs in S18 were predominantly expressed in flowers (Additional file 3: Figure S2) and AtMYBs in the subgroup were involved in anther development [33-41]. Taken together, the results suggest the conserved regulatory mechanism of miRNA-MYB module in flower development in *S. miltiorrhiza* and *Arabidopsis*.

The 38 miRNA-targeted SmMYBs distribute in 21 subgroups (Figures 1 and 5). Targets of miR159 are included in S18 and S36. Targets of miR828 belong to S6 and S15. MiR858-regulated *SmMYBs* widely distribute in 19 subgroups. It suggests the significance of miRNAs in regulating multiple cellular processes. Except S15 and S18, each of other 19 subgroups was regulated by only a miRNA family. It is consistent with the fact that MYBs in a subgroup show high sequence similarity and usually play redundant roles.

### SmMYBs potentially involved in the biosynthesis of bioactive compounds

*S. miltiorrhiza* Bunge is a widely used Chinese medicinal material. The main bioactive compounds in *S. miltiorrhiza* are terpenoid tanshinones and phenolic acids. Various R2R3-MYBs were found to regulate the biosynthesis of terpenoids. For instance, AtMYB62 included in S20 is involved in gibberellin metabolism and signaling and affect root architecture, Pi uptake and acid phosphatase activity [66]. Grapevine VvMYB5b belonging to S5 is involved in flavonoid and terpenoid metabolism. Overexpression of VvMYB5b resulted in the down-regulation of phenylpropanoid metabolism whereas up-regulation of carotenoid metabolism [9]. Loblolly pine PtMYB14 belonging to S4 is a putative regulator of an isoprenoid-oriented response that leads to the accumulation of sesquiterpene in conifers [8]. It implies that SmMYBs belonging to subgroups 4, 5 and 20 are potential regulators of terpenoid biosynthesis in *S. miltiorrhiza*. Since terpenoid biosynthesis-related plant MYBs have not been systematically investigated, it is possible that some SmMYBs in other subgroups are also involved in terpenoid biosynthesis.

Phenolic acids are the other main group of bioactive compounds in *S. miltiorrhiza*. The main pathways leading to the formation of these compounds start with the aromatic amino acids L-phenylalanine and L-tyrosine. So far, a large number of R2R3-MYBs belonging to various subgroups, including S3, S4, S5, S6, S7, S13 and S21, have been found to affect the biosynthesis of these compounds. S3 MYBs, such as AtMYB58 and AtMYB63, were shown to activate *PAL*, *C4H*, *4CL* and various other genes involved in lignin biosynthesis [43]. SmMYB37 belongs to the same subgroup and is predominately expressed in *S. miltiorrhiza* stems (Figure 1 and Additional file 3: Figure S2), implying the role of SmMYB37 in lignin biosynthesis. The other MYBs involved in lignin deposition are members of S13 and S21 [67,68]. S4 includes 6 SmMYBs and 6 AtMYBs (Figure 1). AtMYB4 is a repressor of *C4H*. Down-regulation of AtMYB32 increased transcript levels of *COMT* gene involved in lignin biosynthesis and reduced the levels of *DFR* and *ANS* genes associated with proanthocyanidin biosynthesis [69]. S5 MYBs, AtMYB5 and AtMYB123, are involved in the biosynthesis of proanthocyanidins and tannins in *Arabidopsis* seeds. Additionally, AtMYB5 is also involved in trichome development [61,70]. It indicate the role of 6 SmMYBs (SmMYB3, SmMYB28, SmMYB54, SmMYB76, SmMYB93 and SmMYB109) belonging to S4 and SmMYB6 included in S5 in proanthocyanidin biosynthesis. All of S6 AtMYBs, including AtMYB75, AtMYB90, AtMYB113 and AtMYB114, regulate anthocyanin biosynthesis [28], implying that SmMYB36 belonging to the same subgroup is also involved in anthocyanin biosynthesis. SmMYB97 is clustered into S7 with AtMYB11, AtMYB12 and AtMYB111 controlling flavonoid biosynthesis in *Arabidopsis*[24,71]. Thus, SmMYB97 is a potential regulator of flavonol biosynthesis in *S. miltiorrhiza*. Further analysis of *SmMYBs* through genetic transformation will definitely shed lights on the regulatory mechanism of bioactive compound biosynthesis.

## Conclusions

The *MYB* gene family is the largest transcription factor family in plants. It plays significant roles in plant development, secondary metabolism, and stress responses. The identification and characterization of 110 *SmMYB* genes provides a foundation for understanding the regulatory mechanism of *MYBs* in *S. miltiorrhiza*. Through the comprehensive classification and comparative analysis of the *R2R3*-*MYB* gene family in *S. miltiorrhiza* and *Arabidopsis*, we showed the conservation and diversity of *MYBs* in plants. The distribution of residues in the MYB domain of *S. miltiorrhiza* R2R3-MYBs is quite similar to *Arabidopsis*, although the patterns at various positions are different. A total of 40 motifs were identified in downstream of the MYB domain of SmMYBs and AtMYBs. Among them, 35 exist in both SmMYBs and AtMYBs, 5 are AtMYB-specific. Based on the phylogenetic tree and previous results from *Arabidopsis*[10], SmMYBs and AtMYBs were classified into 37 subgroups, of which 31 include proteins from *S. miltiorrhiza* and *Arabidopsis*, whereas 2 are specific to *S. miltiorrhiza* and 4 are specific to *Arabidopsis*. Many *S. miltiorrhiza* R2R3-MYBs in a subgroup share at least a motif. *SmMYBs* were differentially expressed in roots, stems, leaves and flowers of *S. miltiorrhiza*. The expression profiles are largely consistent with known functions of their *Arabidopsis* counterparts. We identified 38 *SmMYBs* to be targets of miRNAs belonging to the *MIR159*/*R319*, *MIR828* and *MIR858* families. Among them, 36 are regulated by a miRNA family, while the other 2 are co-regulated by miRNAs from two families. The regulatory mechanism of miRNA-MYB module is also conserved in *Arabidopsis*. MiRNA-targeted *SmMYBs* are widely distributed in different subgroups, suggesting the significance of miRNAs in regulating multiple cellular processes. Based on functional conservation of SmMYBs and AtMYBs, we predicted some functions of various SmMYBs. SmMYBs in S4, S5 and S20 are potential regulators of terpenoid biosynthesis, whereas those in S3, S4, S5, S6, S7, S13 and S21 appear to be involved in phenolic acid biosynthesis. Our results suggest the significant and complexity of MYB regulatory networks in *S. miltiorrhiza* and provide useful information for improving the growth and defense abilities and the production of bioactive compounds in *S. miltiorrhiza*.

## Methods

### Genome-wide survey of *S. miltiorrhiza R2R3*-*MYB* genes

The protein sequences of 125 *A. thaliana* R2R3-MYBs described by Stracke *et al*. (2001) were downloaded from the *Arabidopsis* Information Resource (TAIR, http://www.Arabidopsis.org/) (Additional file 4: Table S2) [12]. Using the tBLASTn algorithm [72], *S. miltiorrhiza* genomic sequences putatively encoding *R2R3*-*MYB* genes were identified by BLAST analysis of *A. thaliana* R2R3-MYBs against the current assembly of the *S. miltiorrhiza* genome that was estimated to represent ~92% of the entire genome and ~96% of the protein-coding genes (Chen et al., unpublished). An e-value cut-off of 1e–10 was applied to the homologue recognition. Gene models were predicted as described previously [7]. First, all retrieved genomic sequences of the *S. miltiorrhiza* genome were used for gene prediction on the Genscan web server ((http://genes.mit.edu/GENSCAN.html). Second, the predicted gene models were BLAST-analyzed against the non-redundant protein sequence (nr) database (http://www.ncbi.nlm.nih.gov/BLAST) using the BLASTx algorithm with default parameters. Finally, the gene models were manually corrected after carefully checked the alignment between *SmMYB* genes and *MYBs* from other plant species. The MYB domain of each SmMYB protein was predicted using Pfam 26.0 on the Pfam sever with default parameters (http://pfam.sanger.ac.uk). Protein sequences with two repeats in the MYB domain were recognized as members of the R2R3-MYB subfamily.

### RNA extraction, coding sequence (CDS) cloning and quantitative real-time reverse transcription-PCR (qRT-PCR)

Roots, stems, leaves and flowers of whole genome-decoded *S. miltiorrhiza* Bunge (line 993) were collected and stored in liquid nitrogen until use. Total RNA was extracted using the plant total RNA extraction kit (Aidlab, China). Genomic DNA contamination was eliminated by pre-treating total RNA with RNase-Free DNase (Promega, USA). RNA integrity was analyzed on a 1.2% agarose gel. RNA quantity was determined using a NanoDrop 2000C Spectrophotometer (Thermo Scientific, USA). Total RNA was reverse-transcribed by Superscript III Reverse Transcriptase (Invitrogen, USA). The full-length CDSs of 110 *SmMYBs* were amplified by PCR using the primers listed in Additional file 5: Table S3. PCR products were gel-purified, cloned, and then sequenced. qRT-PCRs were performed as previously described by Ma *et al*. using gene-specific primers (Additional file 6: Table S4) [7]. The length of amplicons was between 80 bp and 250 bp. *SmUBQ10* was used as a reference gene. The data was analyzed as described previously [7].

### Analysis of the MYB domain and other motifs

To analyze the features of MYB domains of *S. miltiorrhiza* R2R3-MYB proteins, the amino acid sequence of R2 and R3 repeats in 110 *S. miltiorrhiza* R2R3-MYB proteins were aligned with the ClustalW method using BioEdit software (http://www.mbio.ncsu.edu/BioEdit/bioedit.html) and adjusted manually. The sequence logos for R2 and R3 MYB repeats were created by submitting the multiple alignment sequences to the WebLogo server (http://weblogo.berkeley.edu/logo.cgi) [73]. Potential protein motifs outside the MYB domain were predicted using the MEME Suite version 3.5.7 [74]. The following parameter settings were applied. It includes the distribution of motifs: zero and one per sequence; maximum number of motifs to find: 40, minimum width of motif: 6, and maximum width of motif: 120. The motif must be present in all members within the same subgroup and only those with an e-value less than 1e–10 were kept for further analysis.

### Phylogenetic tree construction

To generate the phylogenetic trees of R2R3-MYB transcription factor genes, a neighbor-joining (NJ) tree was constructed for the full-length protein sequences of 110 SmMYBs and 125 AtMYBs using MEGA version 4.0. Bootstrapping (1000 replicates) was performed to support the statistical reliability.

### Identification of *SmMYBs* with perfect or near-perfect complementary sequences to miRNAs

Plant miRNA sequences were downloaded from miRBase (http://www.mirbase.org/) [60]. The complementary sequences of *SmMYBs* to miRNAs were searched using psRNATarget [64]. The maximum expectations of 3.0 and the target accessibility-allowed maximum energy to unpair the target site of 50 were applied.

### Availability of supporting data

SmMYB sequences supporting the results of this article are available in GenBank under accession numbers KF059355–KF059464. Phylogenetic data supporting the results of this article are available in the TreeBASE repository, http://purl.org/phylo/treebase/phylows/study/TB2:S15578.

## Abbreviations

4CL: 4-coumarate coenzyme A: ligase; C4H: Cinnamate 4-hydroxylase; CDS: Sequence coding for amino acids in protein; MEP: 2-C-methyl-D-erythritol 4-phosphate; miRNA: microRNA; MVA: Mevalonate; PAL: Phenylalanine ammonia-lyase; qRT-PCR: Quantitative real-time reverse transcription-PCR; TCM: Traditional Chinese medicine.

## Competing interests

The authors declare that they have no competing interests.

## Authors’ contributions

CL contributed to RNA extraction, coding sequence (CDS) cloning, qRT-PCR and bioinformatics analysis, and participated in writing the manuscript. SL designed the experiment, participant in bioinformatics analysis, and wrote the manuscript. Both authors have read and approved the version of manuscript.

## Supplementary Material

Additional file 1: Table S1 Sequence features of *R2R3*-*MYBs* in *S. miltiorrhiza*. Some sequence features of R2R3-MYBs in *S. miltiorrhiza* are shown. Click here for file

Additional file 2: Figure S1 Architecture of conserved protein motifs in SmMYBs and AtMYBs. Conserved motifs are indicated in numbered color boxes. Click here for file

Additional file 3: Figure S2 Expression patterns of *SmMYB* genes in various tissues of *S. miltiorrhiza*. Fold changes of transcript levels in root (RT), stems (St), leaves (Le) and flowers (Fl) of *S. miltiorrhiza* plants are shown. *SmMYBs* expression is relative to SmUBQ10*10000. Click here for file

Additional file 4: Table S2 Sequence features of *R2R3*-*MYBs* in *A. thaliana*. Some sequence features of R2R3-MYBs in *A. thaliana* are shown. Click here for file

Additional file 5: Table S3 Primers used for cloning of *SmMYB* CDSs. Complete set of primers used for amplification of *SmMYB* CDSs. Click here for file

Additional file 6: Table S4 Primers used for qRT-PCR analysis of *SmMYB* genes. Complete set of primers used for qRT-PCR. Click here for file
