# Supplementary material for: Genome-wide characterization and comparative analysis of R2R3-MYB transcription factors shows the complexity of MYB-associated regulatory networks in Salvia miltiorrhiza
Source: BMC Genomics. 2014 Apr 11;15:277. doi: 10.1186/1471-2164-15-277 (PMC4023596; doi:10.1186/1471-2164-15-277)
Supplement: Additional file 2: Figure S1 — Architecture of conserved protein motifs in SmMYBs and AtMYBs. Conserved motifs are indicated in numbered color boxes. [file 1471-2164-15-277-S2.doc]

**Additional file 2.** Architecture of conserved protein motifs in SmMYBs and AtMYBs.
