# Supplementary material for: Genome-wide characterization and comparative analysis of R2R3-MYB transcription factors shows the complexity of MYB-associated regulatory networks in Salvia miltiorrhiza
Source: BMC Genomics. 2014 Apr 11;15:277. doi: 10.1186/1471-2164-15-277 (PMC4023596; doi:10.1186/1471-2164-15-277)
Supplement: Additional file 3: Figure S2 — Expression patterns of SmMYB genes in various tissues of S. miltiorrhiza. Fold changes of transcript levels in root (RT), stems (St), leaves (Le) and flowers (Fl) of S. miltiorrhiza plants are shown. SmMYBs expression is relative to SmUBQ10*10000. [file 1471-2164-15-277-S3.doc]

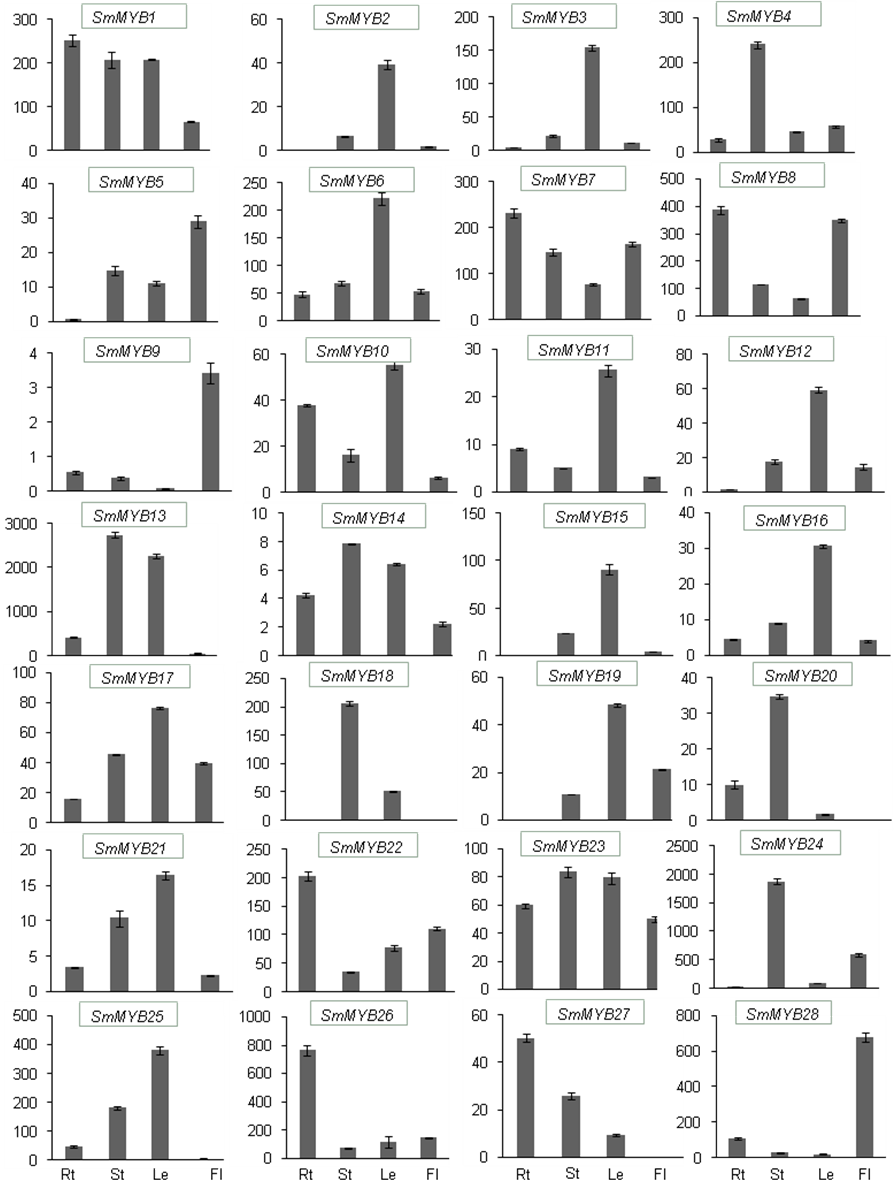


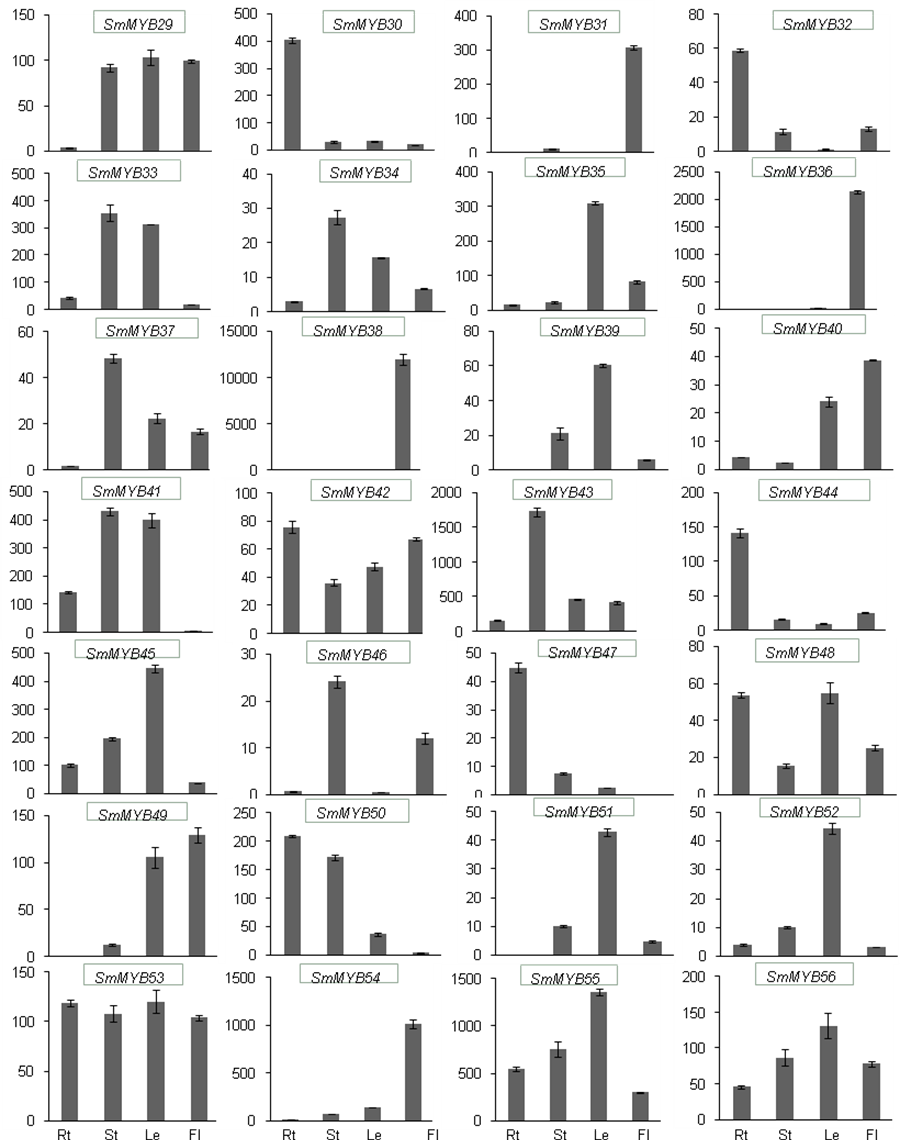


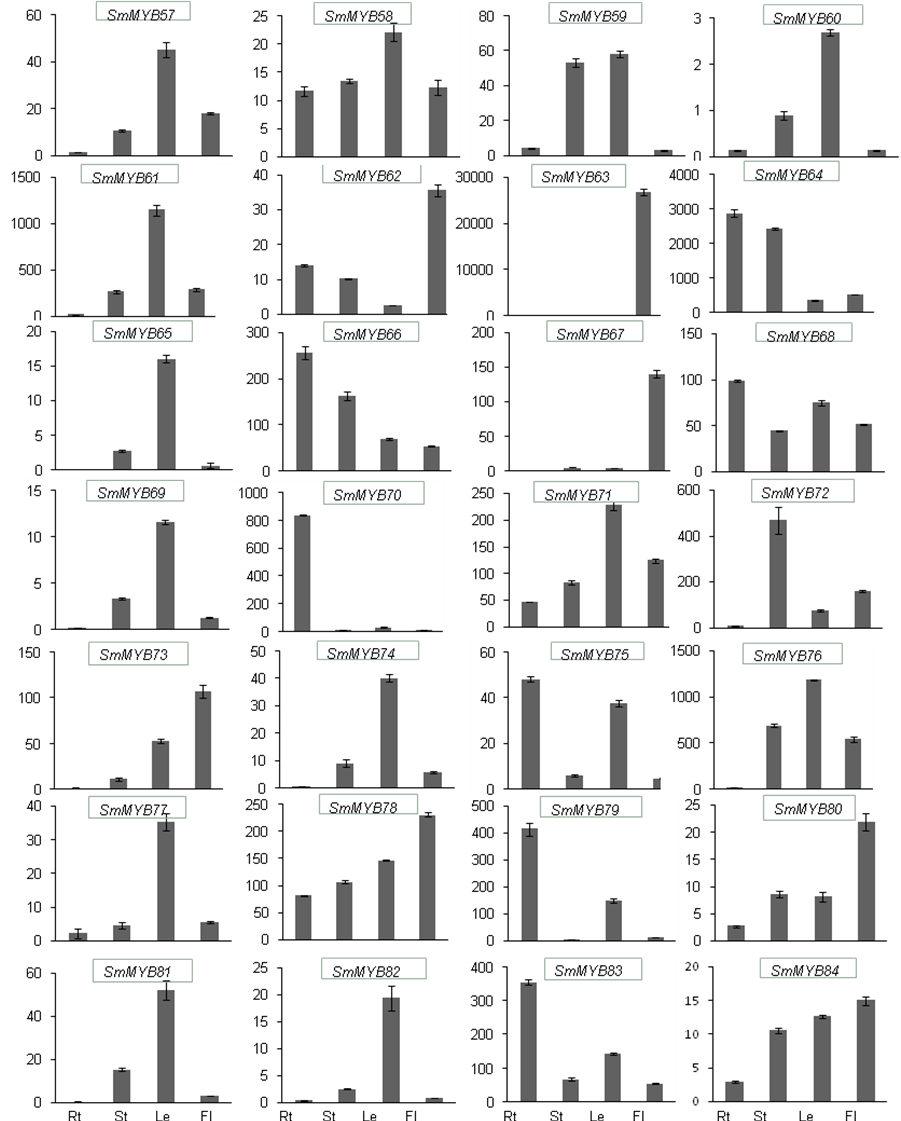


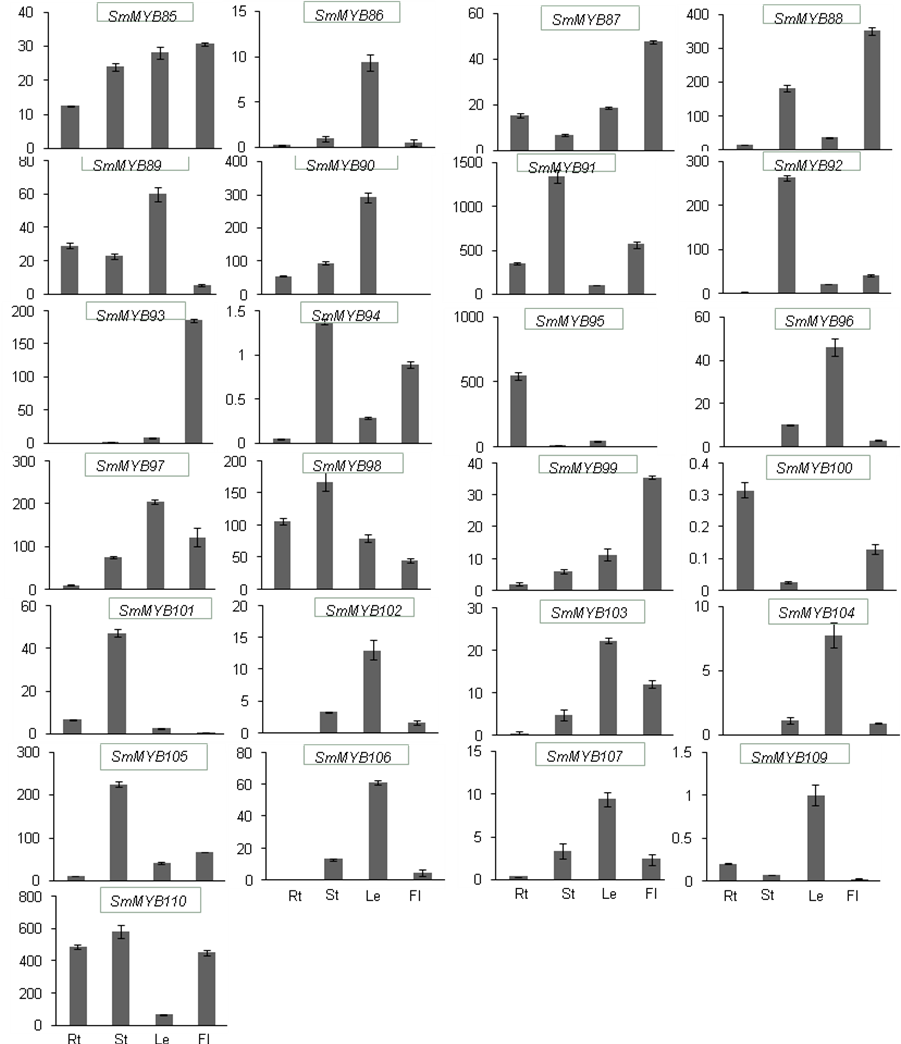


**Additional file 3.** Expression patterns of *SmMYB* genes in various tissues of *S. miltiorrhiza*. Fold changes of transcript levels in root (RT), stems (St), leaves (Le) and flowers (Fl) of *S. miltiorrhiza* plants are shown. *SmMYBs* expression is relative to *SmUBQ10**10000.

(Relative to *SmUBQ10**10000.
